# Cost-Utility of Antimicrobial Prophylaxis for Treatment of Children With Vesicoureteral Reflux

**DOI:** 10.3389/fped.2019.00530

**Published:** 2020-01-10

**Authors:** Nader Shaikh, Vinod Rajakumar, Caitlin G. Peterson, Jillian Gorski, Anastasia Ivanova, Lisa Gravens Muller, Yosuke Miyashita, Kenneth J. Smith, Tej Mattoo, Hans G. Pohl, Ranjiv Mathews, Saul P. Greenfield, Steven G. Docimo, Alejandro Hoberman

**Affiliations:** ^1^Division of General Academic Pediatrics, Children's Hospital of Pittsburgh of UPMC, Pittsburgh, PA, United States; ^2^Department of Pediatrics, University of Pittsburgh School of Medicine, Pittsburgh, PA, United States; ^3^Nephrology, University of Utah Health, Salt Lake City, UT, United States; ^4^Department of Pediatrics, Indiana University, Indianapolis, IN, United States; ^5^Biostatistics, University of North Carolina at Chapel Hill, Chapel Hill, NC, United States; ^6^Division of General Internal Medicine, University of Pittsburgh, Pittsburgh, PA, United States; ^7^Nephrology, Wayne State University School of Medicine, Detroit, MI, United States; ^8^Children's National Medical Center, Washington, DC, United States; ^9^Southern Illinois University School of Medicine, Springfield, IL, United States; ^10^Department of Pediatrics and Urology, Zucker School of Medicine, New York, NY, United States

**Keywords:** cost-effectiveness, cost utility, urinary tract infection (UTI), pediatric, pediatric infectious disease

## Abstract

**Objective:** Antimicrobial prophylaxis for children with vesicoureteral reflux (VUR) reduces recurrences of urinary tract infection (UTI) but requires daily antimicrobials for extended periods. We used a cost-utility model to evaluate whether the benefits of antimicrobial prophylaxis outweigh its risks and, if so, to investigate whether the benefits and risks vary according to grade of VUR.

**Methods:** We compared the cost per quality-adjusted life-year (QALY) gained in four treatment strategies in children aged <6 years diagnosed with VUR after a first UTI, considering these treatment strategies: (1) prophylaxis for all children with VUR, (2) prophylaxis for children with Grade III or Grade IV VUR, (3) prophylaxis for children with Grade IV VUR, and (4) no prophylaxis. Costs and effectiveness were estimated over the patient's lifetime. We used $100,000/QALY gained as the threshold for considering a treatment strategy cost effective.

**Results:** Based on current data and plausible ranges to account for data uncertainty, prophylaxis of children with Grades IV VUR costs $37,903 per QALY gained. Treating children with Grade III and IV VUR costs an additional $302,024 per QALY gained. Treating children with all grades of VUR costs an additional $339,740 per QALY gained.

**Conclusions:** Treating children with Grades I, II, and III VUR with long-term antimicrobial prophylaxis costs substantially more than interventions typically considered economically reasonable. Prophylaxis in children with Grade IV VUR is cost effective.

## Background

Although long-term antimicrobial prophylaxis for children with VUR has been shown to substantially reduce recurrences of UTI (from 27.4 to 14.8% during a 2-year follow-up period) (1), its use in children with VUR remains contentious. Proponents argue that, by preventing recurrent febrile UTIs, antimicrobial prophylaxis has the potential to reduce subsequent hypertension (HTN), preeclampsia, chronic kidney disease (CKD), and end-stage renal disease (ESRD). Opponents argue that the risk of prophylaxis outweighs its benefits, because, using the aforementioned data, only 27.4% of children with untreated VUR developed a reinfection during the 2-year follow-up period. Others argue that the benefits of treatment may differ in children according to the grade of VUR.

A cost-utility analysis is particularly well-suited to address this controversy, because both risks and benefits can be combined into a single metric, cost per quality-adjusted life year (QALY) gained and compared across treatment strategies.

We conducted a cost-utility analysis to evaluate whether the benefits of antimicrobial prophylaxis outweigh its risks in children with known VUR and, if not, for which subgroups of children could prophylaxis be considered cost-effective.

## Patients and Methods

### Overview of the Decision-Analytic Model

We compared clinical and economic outcomes of four treatment strategies in a hypothetical cohort of symptomatic children younger than 6 years of age diagnosed with VUR after a first UTI. Children were assigned to 1 of the following 4 strategies. These strategies include treating all children, leaving all children untreated, and treating only children with high-grade VUR. Some experts consider grades III and IV VUR as being high-grade, others consider only grade IV as high grade. As such we analyzed the data both ways (treat only children grade IV vs. treat both grade III and IV). Although other combinations are possible (e.g., treat grade I and IV), these are not logical because risk or reinfection, and thus renal scarring, increases steadily with increasing grade of VUR. Thus, in our modeling, we considered all logical combinations of VUR grade. Each strategy is described in more detail below. As recommended by the Panel on Cost-Effectiveness in Health and Medicine (2), we adopted a societal perspective and included both direct and indirect medical costs in our model. We constructed and analyzed our decision tree using TreeAge Pro software (TreeAge Software, Williamstown, Massachusetts).

#### Time Horizon

Because long-term sequelae of VUR extend throughout life, we used a time horizon of 78 years, the current average US life expectancy at birth in the United States. We assumed that ESRD would start at age 17 (median age of ESRD in children with reflux nephropathy in the US) (3), and would continue for the patient's lifetime. We assumed that patients with ESRD would have a 7-year period with CKD (4). We also assumed that the life span of an individual with HTN would be 73 years (5).

#### Structure of the Decision Tree

Figure 1s shows the structure of the decision tree. Short term outcomes (outcomes within the first 4 years) included recurrences of UTI and surgery for VUR. Long term outcomes included HTN, preeclampsia, CKD, and ESRD. We chose a decision tree model (rather than a Markov model) because pertinent events occurred over a relatively short time frame (4 years) and these events during this period, albeit recurrent, could be compactly modeled in a decision tree structure. Once pertinent events occur, the remaining costs and QALYs were assigned based on the presence or absence of renal scarring (which had its onset within the relevant time frame).

#### Description of Each Strategy in the Absence of Frequent Febrile Reinfections

Prophylaxis for all children with VUR—all children were started on antimicrobial prophylaxis and continued on prophylaxis for 2 years (in all strategies involving prophylaxis, we assumed that trimethoprim sulfamethoxazole at 2 mg/kg/day would be used for prophylaxis), at which time a VCUG was repeated. If VUR of any grade was still present on the follow-up VCUG, 2 more years of prophylaxis were prescribed, otherwise, children were observed for an additional 2 years without prophylaxis.

Prophylaxis for children with Grade III or Grade IV VUR—only children with grades III or IV VUR were started on antimicrobial prophylaxis. These children received prophylaxis for 2 years at which time a VCUG was repeated (a repeat VCUG was not performed on children with Grade I or II VUR). If grades III or IV VUR was still present on the follow-up VCUG, 2 more years of prophylaxis were prescribed; children with no VUR or grades I, or II VUR at the time of follow-up VCUG were observed for an additional 2 years without prophylaxis.

Prophylaxis for children with Grade IV VUR—only children with grades IV VUR were started on antimicrobial prophylaxis. These children received prophylaxis for 2 years at which time a VCUG was repeated (a repeat VCUG was not performed on children with grade I or II or III VUR). If grade IV VUR was still present on the follow-up VCUG, 2 more years of prophylaxis were prescribed; children with no VUR or grades I, or II or III VUR at the time of follow-up VCUG were observed for an additional 2 years without prophylaxis.

No prophylaxis—all children were observed without antimicrobial prophylaxis for 4 years. No follow-up VCUGs were performed.

#### Changes to the Assigned Strategy for Children With Frequent Febrile Reinfections

Regardless of the initial strategy, children with 2 febrile reinfections who were not on prophylactic antibiotics were started on prophylactic antibiotics. Ureteral reimplantation during the follow-up period was at the discretion of the treating urologist. For the latter, we used individual patient data from the Randomized Intervention for Children with Vesicoureteral Reflux (RIVUR) trial (Table 1s).

### Outcomes

The primary short-term outcome of interest was the development of renal scars during the first 4 years after diagnosis of VUR. The likelihood of renal scarring was estimated from the number of febrile UTIs observed during this period (6). We chose this strategy because the RIVUR trial was not powered to detect small differences in rates of renal scarring. We chose a 4-year time period because significant VUR would have resolved or would have been repaired for most children. Accordingly, after year 4, we assumed that rates of recurrent UTI would be equivalent across all strategies. The number of febrile UTIs in each strategy was obtained from individual-patient data from the RIVUR study, which compared the efficacy of trimethoprim-sulfamethoxazole prophylaxis to placebo in preventing recurrent UTIs (1). That study was well-suited for the present purpose because it included a placebo arm, and short-term outcomes were carefully monitored during the 2-year follow-up period. To allow us to focus this paper on children with a first UTI, in whom antimicrobial treatment remains controversial, children with a history of previous UTIs before enrollment into the RIVUR study were excluded. Because the incidence of UTI decreased by 50% from year 1 to 2 in the RIVUR study, we estimated that in years 3 and 4 we would observe half as many UTIs as we observed in year 2 of the RIVUR study.

The primary long-term outcomes of interest were HTN, preeclampsia, CKD, and ESRD. We used the estimated rate of renal scarring in the first 4 years in each strategy (which was largely determined using individual patient data) to estimate the probability of long-term outcomes. For the latter we used data from published studies that reported incidences of these outcomes in children with renal scarring; children without renal scarring were assumed not to have long-term sequelae attributable to UTIs. All children with ESRD were assumed to have had CKD for an average of 7.4 years before the need for dialysis (7).

### Base-Case Estimates of Costs and Values

Values used in the model for costs and quality-of-life utilities associated with individual clinical states are summarized in Table 1.

**Table 1:** Costs (before discounting), quality adjusted years lost (before discounting), and probabilities associated with specific clinical states, as used in the decision model.

**Clinical states**	**Best estimate**	**Plausible range^**a**^**	**References**
**Costs ($)**			
Trimethoprim-sulfamethoxazole (annual)	645	323–968	Red Book (8)
3-day hospital admission^b^	5,346	2,673–8,019	HCUP (9), CMS (10), BLS (11)
Emergency room visit^c^	265	133–398	CMS (10), BLS (11)
Office visit for UTI^d^	191	96–287	CMS (10), BLS (11)
Urinalysis	4	2–7	DHHS (12)
Urine culture	11	6–17	DHHS (12)
Cefdinir 10 day course	51	25–76	Red Book (8)
Cephalexin 10 day course	32	16–47	Red Book (8)
Surgery^e^	7,772	3,886–11,658	Lackgren (13)
Voiding cystourethrogram^f^	373	187–560	CMS (10)
Preeclampsia (each episode)^g^	9,200	4,600–13,800	UCLA CHPR (14)
HTN (annual)	1,131	566–1,697	Balu and Thomas (15)
CKD (annual)	5,736	2,868–8,604	CDC (16), Honeycutt et al. (17)
Hemodialysis for end stage renal disease (annual)	84,550	42,275–126,825	USRDS (3)
Peritoneal dialysis for end stage renal disease (annual)	69,919	34,960–104,879	USRDS (3)
Transplant for end stage renal disease (annual)	29,920	14,960–44,880	USRDS (3)
**Quality adjusted life years lost**^**h**^			
Daily trimethoprim sulfamethoxazole^i^	0.000694	0.00–0.0069	Estimate
Febrile UTI (per episode)^j^	0.0072	0.0052–0.0090	Barry et al. (18)
Surgery (per operation)	0.05	0.00–0.15	Hsieh et al. (19)
Voiding cystourethrogram (per scan)^k^	0.000228	0.00–0.00046	Estimate
Preeclampsia (each episode)	0.08	0.00–0.18	Sonnenberg et al. (20), CDC (16)
Hypertension	0.10	0.00–0.20	Estimate
Chronic kidney disease	0.41	0.31–0.51	Martinell et al. (21), Tong et al. (22)
Hemodialysis for end stage renal disease	0.56	0.46–0.66	Lee (4)
Peritoneal dialysis for end stage renal disease	0.43	0.33–0.53	Lee (4)
Transplant for end stage renal disease	0.29	0.19–0.39	Lee (4)
**Probability of long-term outcomes**			
Preeclampsia if renal scar^l^	0.11	0.05–0.16	Martinell et al. (21), Smellie et al. (23)
Hypertension if renal scar	0.10	0.05–0.15	Kramer et al. (24)
End stage renal disease or CKD if renal scar	0.0002525	0.0000087–0.01	Round et al. (25), Calderon-Margalit et al. (26)
Hemodialysis (annual) if end stage renal disease	0.52	0.26–0.78	USRDS (3)
Peritoneal dialysis (annual) if end stage renal disease	0.27	0.13–0.40	USRDS (3)
Transplant (annual) if end stage renal disease	0.22	0.11–0.33	USRDS (3)

*HCUP, Healthcare Costs and Utilization Project; CMS, Centers for Medicare/Medicaid Services; BLS, Bureau of Labor Statistics*.

a *Unless otherwise indicated, used 50–150% of best estimate for costs and ±0.1 for utility values*.

b Includes missed work ($21.00/h*8 h*3 days) and parking/transportation ($20.00).

c Includes missed work ($21.00/h*3 h) and parking/transportation ($20.00).

d Includes missed work ($21.00/h*6 h) and parking/transportation ($20.00).

e *Adjusted to 2018 values using the Consumer Price Index*.

f Includes missed work ($21.00/h*4 h) and parking/transportation ($20.00).

g *Assumed 2 pregnancies at ages 26 and 29 (16)*.

h *Per year unless otherwise stated*.

i *Utility of 0 for 1 min per day (range 0–10 min)*.

j *Disutility of 0.37 per day for 1 week*.

k *Utility of 0 for 2 h (range 0–4 h)*.

l *Absolute risk of preeclampsia 11 and 12% higher in women with renal scarring compared to women without renal scarring in the Martinell and Smellie studies, respectively*.

#### Costs

##### Healthcare costs

Healthcare costs included costs of visits, medications, and laboratory tests. Accordingly, for each recurrent UTI, we included costs of the physician visit, antibiotics (cefdinir for 10 days in children on prophylaxis and cephalexin for 10 days for children not on prophylaxis), urine culture, and urinalysis.

Because HTN, preeclampsia, CKD, and ESRD occur years after the index UTI, future costs associated with these states were discounted at a rate of 3% per year, in accordance with US recommendations (2).

##### Non-healthcare costs

For each visit, we included non-medical costs. These included cost of work lost by parents, cost of childcare if needed, and cost of parking. We estimated the cost of work lost by multiplying estimated hours of work missed (27) by average wage as reported by the U.S. Bureau of Labor Statistics (11).

#### Utility Values and Quality of Life

Utility reflects the health-related quality of life of an individual at a particular point in time. Utility values are scaled from 0 to 1, where 0 denotes death and 1 denotes perfect health. Disutility values are one minus the utility values. For each undesirable state, we determined the QALYs lost by multiplying the time spent in that state by its associated disutility value. Similar to costs, we discounted QALYs at a rate of 3% annually. Of note, because VUR is an asymptomatic condition, no disutility value was assigned to it in our models. Rather, to arrive at the disutility values in each strategy, we relied on the number of symptomatic events each child with VUR experienced (e.g., UTIs, VCUGs).

### Impact of Alternate Treatment Strategies

For the main analysis, we assumed, as per current standard practice (28), that management after the initial 2-year period, would be largely driven by the grade of VUR at the time of repeat VCUG. Recently, some have proposed that, rather than using VUR grade, occurrence of febrile UTI after diagnosis of VUR (29), should guide subsequent management (i.e., no repeat VCUG performed if no breakthrough UTIs). Accordingly, to test the robustness of our model, we constructed a model in which, after 2 years of the initial treatment strategy, antimicrobial prophylaxis was continued for another 2 years only in children receiving antimicrobial prophylaxis who had at least 1 febrile recurrence in the first 2 years after diagnosis.

### Analyses

We compared strategies using their incremental cost-utility ratios (ICERs), defined as the extra cost of a more expensive strategy compared to the cost of the nearest less expensive strategy, divided by the added QALYs conferred by the more expensive strategy. We used a $100,000 per QALY gained, a commonly cited US benchmark (30), as our cost-effectiveness willingness-to-pay threshold. We conducted one-way sensitivity analyses for variables by varying baseline estimates within clinically plausible ranges. Whenever individual patient data were available, we based ranges on 95% confidence intervals calculated using the Clopper-Pearson formula. We also conducted probabilistic sensitivity analyses in which we varied all parameters simultaneously over distributions, using beta distributions for probabilities and utilities and gamma distributions for costs, with distributions fitted to approximate ranges in Table 1. In these analyses, values from each probability distribution are randomly selected during each of 10,000 iterations, and the percentage of iterations for which a given strategy was favored was tracked.

## Results

Table 2 shows cost-effectiveness analysis results for each of the four treatment options. The total cost of each treatment strategy, listed in order from most to least costly, was as follows: prophylaxis for all children, prophylaxis for children with Grade III or IV VUR, prophylaxis for children with Grade IV VUR, and no prophylaxis for any child. The high cost of non-cost-effective strategies was mainly driven by the higher proportion of children requiring prophylaxis in these strategies (Table 2).

**Table 2:** Comparative cost-utility of 4 treatment strategies for treatment of vesicoureteral reflux in children <6 years of age.

**Analytic components**	**Treatment strategy**^**a**^			
	**No prophylaxis regardless of grade**	**Prophylaxis for VUR IV only**	**Prophylaxis for VUR III or IV only**	**Prophylaxis for all grades**
**Average cost ($) per child of …**				
Recurrent urinary tract infections	243.36	226.06	187.71	106.80
Antimicrobial prophylaxis	0	119.84	742.01	1959.99
Voiding cystourethrogram	0	29.69	173.33	373.00
Surgery for vesicoureteral reflux	419.69	358.94	247.37	169.42
Treatment of preeclampsia	65.74	62.14	56.97	45.10
Treatment of hypertension	164.21	155.22	142.30	112.66
Treatment of chronic kidney disease	0.73	0.69	0.63	0.50
Treatment of end stage renal disease	12.22	11.56	10.59	8.39
Total	905.95	964.14	1560.91	2775.87
**Average QALYs lost per child's lifetime to…**				
Recurrent urinary tract infection	0.0028	0.0026	0.0021	0.0012
Taking antimicrobial prophylaxis	0.0000	0.0001	0.0008	0.0021
Voiding cystourethrogram	0.0000	0.0000	0.0001	0.0002
Surgery for vesicoureteral reflux	0.0027	0.0023	0.0016	0.0011
Treatment of preeclampsia	0.0006	0.0006	0.0005	0.0004
Treatment of hypertension	0.0191	0.0180	0.0165	0.0131
Treatment of chronic kidney disease	0.0000	0.0000	0.0000	0.0000
Treatment of end stage renal disease	0.0001	0.0001	0.0001	0.0001
Total	0.0253	0.0238	0.0218	0.0182
**Incremental cost-utility ratio**^**b**^	N/A	37,903	303,024	339,740

a *The strategies are listed in order from least expensive to most expensive*.

b *ICER compares each treatment strategy with next most effective strategy*.

The effectiveness of each strategy, listed from most effective to least effective, was as follows: prophylaxis for all children with VUR, prophylaxis for children with Grade III or IV VUR, prophylaxis for children with Grade IV VUR, and no prophylaxis for any child. The low effectiveness of no prophylaxis was largely driven by the higher rates of surgery relative to the other strategies (Table 2). No prophylaxis for any child was the least costly and also the least effective treatment strategy. Prophylaxis for all Grades of VUR was the most costly and most effective treatment strategy. Prophylaxis only for children with Grade IV VUR compared to not using prophylaxis on any child cost $37,903 per QALY gained (Table 2), while prescribing prophylaxis for Grades III and IV compared with prescribing prophylaxis only for children with Grade IV VUR cost an additional $302,024 per QALY gained. Results of the probabilistic sensitivity analysis were consistent with the base case analysis, showing that prophylaxis of Grade IV VUR was the strategy most likely to be favored if willingness-to-pay thresholds are $40,000/QALY gained or more, with no prophylaxis favored at lower thresholds and other strategies unlikely to be favored at any threshold considered (Figure 2s).

Table 3 shows the number of children with HTN, preeclampsia, and ESRD in each treatment strategy for 1,000,000 children entered into the model. In more effective strategies, fewer children developed long term sequelae; for example, all prophylaxis (the most effective strategy) had the lowest number of cases of HTN, preeclampsia, and ESRD.

**Table 3:** Number of children with each long-term sequela according to treatment strategy in a population of 1,000,000 children with known vesicoureteral reflux.

**Long-term outcomes**	**Treatment strategy**			
	**No Prophylaxis regardless of Grade**	**Prophylaxis for VUR IV only**	**Prophylaxis for VUR III or IV only**	**Prophylaxis for all Grades**
No. with preeclampsia	9,438	8,921	8,184	6,479
No. with hypertension	8,580	8,110	7,440	5,890
No. with chronic/end-stage renal disease	22	20	19	15
Incremental cost of treatment ($)^a^	N/A	58,185,539	596,778,864	1,214,960,610

a *Cost of strategy in addition to cost needed for next lest costly strategy with next most effective strategy*.

We conducted one-way sensitivity analyses to identify variables that would change the preferred strategy, using the $100,000/QALY gained criterion, from prophylaxis for only children with VUR IV to any of the other strategies. Only one parameter did so (disutility of prophylaxis, Table 2s). In no case did strategies that included treatment of children with lower grades of VUR become preferred.

Results were similar when using the alternate treatment strategy mentioned above: treatment of grade IV VUR was the most cost-effective strategy and its ICER was ($43,798/QALY gained) similar to what we found in our initially considered strategy.

## Discussion

We sought to determine whether immediate antimicrobial therapy, given its benefits, adverse effects, and costs, is cost-effective in children younger than 6 years of age with VUR and, if so, for which subgroup of children antimicrobial therapy is most cost-effective.

Of the four strategies we considered, we found that prophylaxis for only Grade IV VUR was the most cost-effective with an ICER of $37,903 per QALY gained compared to the “no prophylaxis” strategy, which is < $100,000 per QALY gained that is generally considered cost-effective (30). Of note, our main findings would have been the same even if we had chosen another frequently used threshold (i.e., $50,000 or $200,000) (31–33). The results of sensitivity analyses indicated that the findings were robust; only one variable (disutility of taking daily prophylactic medications) flipped the preferred strategy (from “prophylaxis for Grade IV VUR” to “no prophylaxis”). In contrast, the two strategies which involved treatment of children with low-grade VUR (prophylaxis for all children with VUR, or prophylaxis for children with Grades III or IV VUR) never approached the cost-effectiveness threshold in sensitivity analyses. Our results suggest that use of long-term antimicrobial prophylaxis for children with Grades I–III VUR is unlikely to be considered economically reasonable given the data used and the assumptions made. Reduction of antibiotic use for children with VUR, most of whom have low-grade VUR, constitutes an important contribution to public health.

A strength of our study was our use of individual patient data to obtain values for most parameters. Our study had several limitations. We did not consider children with Grade V VUR because such children were not included in the RIVUR study. Nor did we consider the potential impact of antimicrobial-induced bacterial resistance; however, considering it would likely further strengthen our conclusion that treating children with low-grade VUR is not cost-effective. Because most of our data came from the RIVUR study, the conclusions are only readily applicable to patients in the United States. Furthermore, we chose to use the definitions of “treatment failure” used in the RIVUR study (no change in treatment strategy unless two febrile reinfections). There are countless other ways of defining “treatment failure” in current practice; however, modeling these strategies would require making assumptions regarding the number of reinfections occurring in each of these hypothetical scenarios. In order to limit the number of assumptions made, we used the definition that the RIVUR study investigators agreed upon at the time as a clinically reasonable strategy for managing children with VUR. Accordingly, our results need to be interpreted in this context. To check the robustness of our analysis we chose to examine the results if we had chosen an alternate (and very different) strategy for the management of children after the initial 2-year period; that the results were largely similar is reassuring.

As with any modeling study, conclusions are limited by the available data. Long-term risks of HTN and ESRD failure resulting from UTI-related renal scarring are highly uncertain. However, in sensitivity analyses, we found that variation of ESRD risk within the reported range did not affect strategy favorability, while lower HTN risk could shift favorability to the no prophylaxis strategy; the analysis was insensitive to higher levels of hypertension risk from scarring. From among a long list of possible variables, we chose to stratify our analyses based on grade of VUR. We did so because VUR is a known risk factor for renal scarring and because it was available for all children included. Furthermore, the relationship between grade of VUR, febrile reinfections, and renal scarring had not been explored in detail in previous manuscripts on the RIVUR study. Although presence of bowel and bladder dysfunction in toilet trained children has also been linked to higher rates of UTI recurrence, most of the children included were not toilet trained: BBD could only be assessed in a minority (18%) of the included children. Accordingly, we did not feel that the available data lent itself readily to a detailed exploration of the role of BBD. Similarly, the proportion of males in RIVUR was very small (9% of the sample were males). Thus, neither sex nor circumcision status could be meaningfully explored using available data. It is important to note that our choice of stratification variable does not mean that the effect of other variables is being ignored in the analyses. On the contrary, because we used individual patient data, the rates of renal scarring within each stratum continues to reflect all the other known and unknown risk factors of recurrent UTIs and renal scarring. We did not assess the cost-effectiveness of endoscopic treatment because the RIVUR trial did not evaluate this intervention and thus no individual patient data were available. Because we used individual patient data from the RIVUR trial the conclusions are most applicable to the types of children enrolled in the RIVUR trial. However, because patients in RIVUR were enrolled from 19 sites and from both primary care and subspecialty clinics, the included data is likely a reasonable representation of the general population with VUR.

In summary, for children <6 years of age with VUR, use of long-term antimicrobial prophylaxis for children with low-grade VUR does not appear to be cost-effective.

## Data Availability Statement

The datasets generated for this study can be found in https://repository.niddk.nih.gov/studies/rivur/?query=RIVUR; https://repository.niddk.nih.gov/studies/cutie/?query=CUTIE.

## Ethics Statement

The studies involving human participants were reviewed and approved by the participating IRBs (University of Pittsburgh IRB, University of Pennsylvania IRB, University of Buffalo IRB, Children's National Human Research Protection Program/IRB, Wayne State University IRB). Written informed consent to participate in this study was provided by the participants' legal guardian/next of kin.

## Author Contributions

NS conceptualized and designed the study, assisted with the analysis, drafted the initial manuscript, and approved the final manuscript as submitted. VR, JG, and CP carried out the analyses, reviewed, and revised the manuscript and approved the final manuscript as submitted. LG and AI performed statistical analyses that were used as inputs for the model and approved the final manuscript as submitted. KS helped to develop the decision tree and approved the final manuscript as submitted. RM assisted with data collection, interpretation of the findings, and approval of the final manuscript. HP, TM, SG, SD, YM, and AH assisted with the interpretation of the findings and approved the final manuscript. All authors contributed substantially to the content of the article, critically revised the article for important intellectual content, approved the final manuscript as submitted, and agree to be accountable for all aspects of the work.

### Table of Contents Summary

Treating children with Grades I, II, and III VUR with long-term antimicrobial prophylaxis costs substantially more than interventions typically considered economically reasonable. Prophylaxis in children with Grade IV VUR could be cost effective.

### What's Known on this Subject

Long-term antimicrobial prophylaxis reduces the incidence of febrile urinary tract infections in children with vesicoureteral reflux (VUR). However, whether risks and costs of antimicrobial therapy outweigh its benefits has not been adequately studied.

### What This Study Adds

Use of antimicrobial prophylaxis appears to be cost effective for children with Grade IV VUR but not for children lower grades of VUR (grades I–III).

### Conflict of Interest

The authors declare that the research was conducted in the absence of any commercial or financial relationships that could be construed as a potential conflict of interest.

## Abbreviations

VUR: vesicoureteral reflux
QALY: quality-adjusted life-year
UTI: urinary tract infection
CKD: chronic kidney disease
ESRD: end-stage renal disease
ICER: incremental cost-utility ratio.
