# Supplementary material for: Cost-Utility of Antimicrobial Prophylaxis for Treatment of Children With Vesicoureteral Reflux
Source: Front Pediatr. 2020 Jan 10;7:530. doi: 10.3389/fped.2019.00530 (PMC6965145; doi:10.3389/fped.2019.00530)

Supplementary Material

Table 1s. Probabilities used in the model that were obtained from the raw data from the RIVUR[1] and CUTIE[2] studies

Probabilities used in the model	Best estimate	Plausible range[a]	Reference/Source
Scarring with 1 febrile UTI	0.03	0.01 – 0.05	RIVUR, CUTIE
Scarring with 2 febrile UTIs	0.26	0.11 – 0.40	RIVUR, CUTIE
Scarring with 3 febrile UTIs	0.29	0.05 – 0.52	RIVUR, CUTIE
Scarring with 4 febrile UTIs[b]	0.50	0.30 – 0.70	Jodal[3]
Scarring with 5 febrile UTIs[b]	0.75	0.55 – 0.95	Jodal[3]
Surgery in "all prophylaxis" strategy	0.02	0.00 – 0.04	RIVUR
Surgery in "no prophylaxis" strategy	0.05	0.03 – 0.08	RIVUR
Surgery in "VUR III-IV prophylaxis" strategy	0.03	0.00 – 0.06	RIVUR
Surgery in "VUR IV prophylaxis" strategy	0.05	0.00 – 0.17	RIVUR
≥1 febrile UTI in "all prophylaxis" strategy in 4 years after diagnosis	0.09	0.06 – 0.13	RIVUR
≥1 febrile UTI in "no prophylaxis" strategy in 4 years after diagnosis	0.19	0.14 – 0.24	RIVUR
≥1 febrile UTI in "VUR III-IV prophylaxis" strategy in 4 years after diagnosis	0.14	0.08 – 0.21	RIVUR
≥1 febrile UTI in "VUR IV prophylaxis" strategy in 4 years after diagnosis	0.17	0.12 – 0.24	RIVUR
Prophylaxis in years 3 and 4 in "all prophylaxis" strategy	0.52	0.48 – 0.56	RIVUR
Prophylaxis in years 3 and 4 in "no prophylaxis" strategy	0	N/A	RIVUR
Prophylaxis in years 3 and 4 in "VUR III-IV prophylaxis" strategy	0.11	0.08 – 0.14	RIVUR
Prophylaxis in years 3 and 4 in "VUR IV prophylaxis" strategy	0.01	0.00 – 0.02	RIVUR
3-day hospitalization if UTI	0.07	0.05 – 0.09	RIVUR
Emergency room visit if UTI	0.43	0.39 – 0.47	RIVUR
Office visit if UTI	0.50	0.46 – 0.54	RIVUR
Proportion of children with VUR I-IV that have VUR IV	0.08	0.06 – 0.10	RIVUR
Proportion of children with VUR I-IV that have VUR III or IV	0.47	0.42 – 0.51	RIVUR
Proportion of included children that are females	0.89	0.87 – 0.92	RIVUR

[a]Used Clopper Pearson formula to estimate plausible range when individual patient data was available
[b]Estimated from Jodal article which reported 58% risk of renal scarring in children with ≥4 febrile UTIs

1. Hoberman A, Greenfield SP, Mattoo TK, et al. Antimicrobial prophylaxis for children with vesicoureteral reflux. *The New England journal of medicine.* 2014;370(25):2367-2376.
2. Keren R, Shaikh N, Pohl H, et al. Risk Factors for Recurrent Urinary Tract Infection and Renal Scarring. *Pediatrics.* 2015;136(1):e13-21.
3. Jodal U. The natural history of bacteriuria in childhood. *Infect Dis Clin North Am.* 1987;1(4):713-729.

Table 2s. Variable from table 1 that was influential in the sensitivity analysis

Variable	Base-Case Value	Threshold Value	Preferred strategy for values below threshold value	Preferred strategy for values above threshold value
QALYs lost for prophylaxis	0.000694	0.006	Grade IV Prophylaxis	No Prophylaxis

Figure 1s. Simplified structure of the decision tree

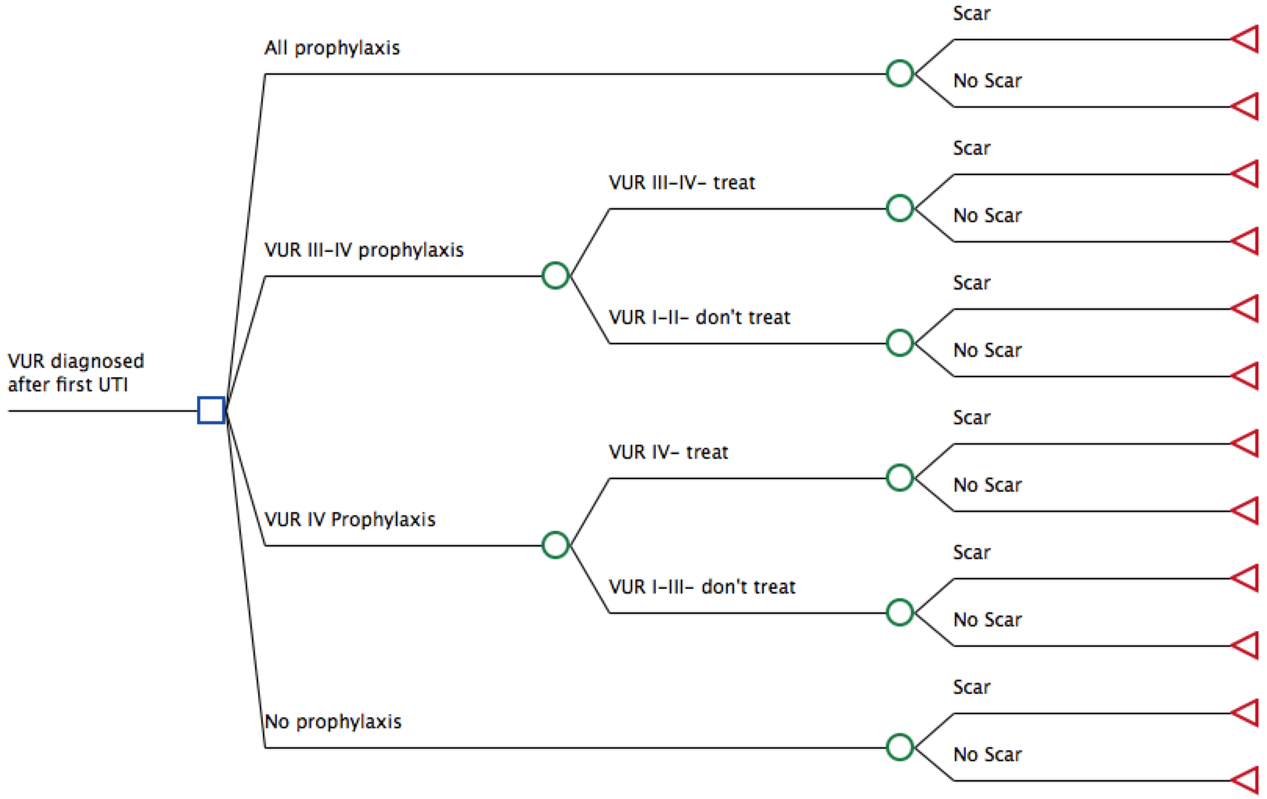

Figure 2s. Probabilistic sensitivity analysis in which the program iteratively selects random values from the distributions of every variable in the model and calculates cost-utility values for each iteration

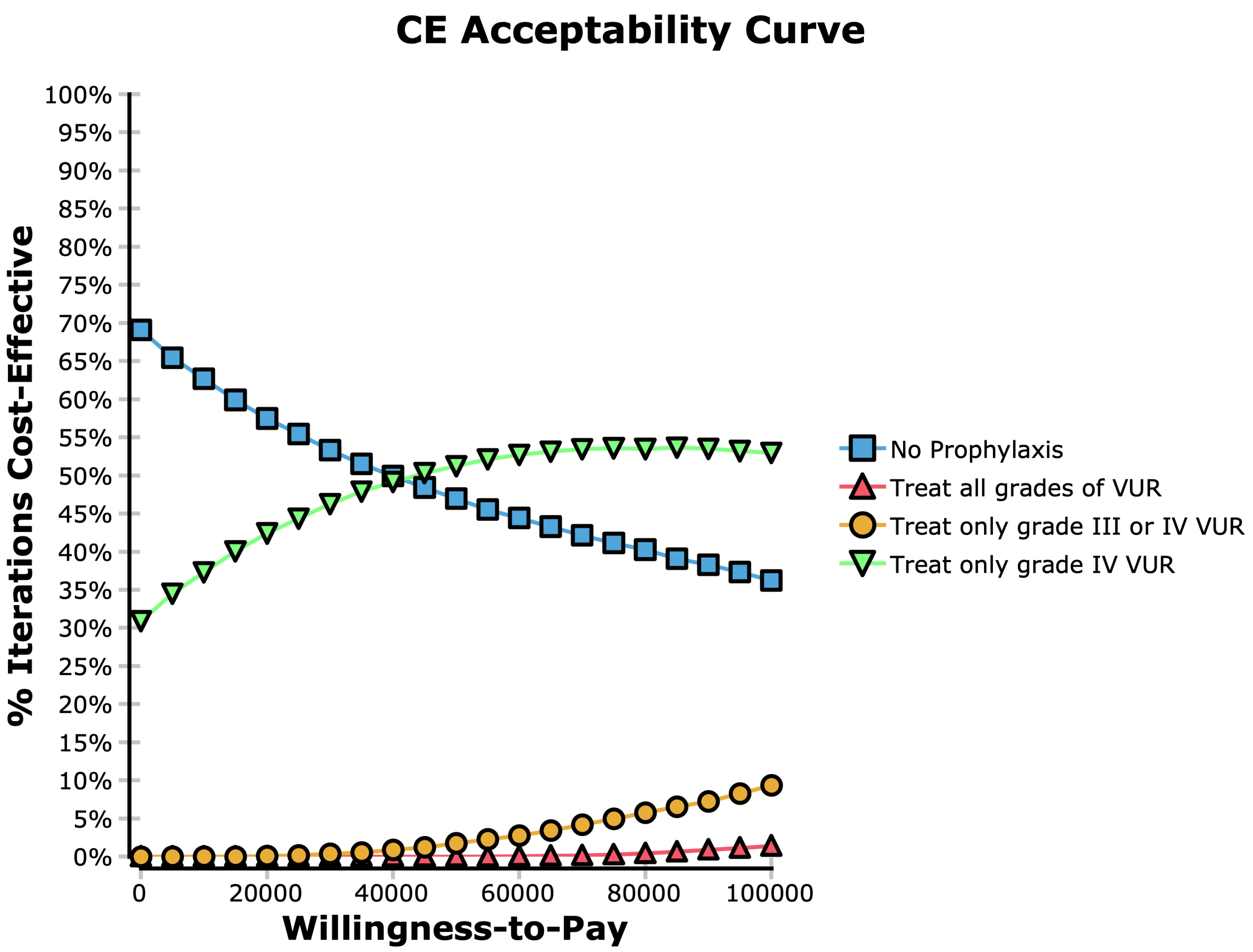

Supplement: Supplementary file 1 [file Presentation_1.pdf]
